# Exosomal miR-486-5p derived from human placental microvascular endothelial cells regulates proliferation and invasion of trophoblasts via targeting IGF1

**DOI:** 10.1007/s13577-021-00543-x

**Published:** 2021-05-11

**Authors:** Ruixia Ma, Zhijiang Liang, Xiaomei Shi, Linli Xu, Xiaowei Li, Jinhua Wu, Lina Zhao, Guocheng Liu

**Affiliations:** Department of Obstetrics, Guangdong Woman and Children Hospital, No. 521 Xingnan Avenue, Guangzhou, 510000 Guangdong China

**Keywords:** PE, Exosomes, HPVEC, MiR-486-5p

## Abstract

Preeclampsia (PE) is a serious complication of pregnancy. Exosomes are known to be upregulated in PE. In this study, we sought to investigate the effect of miR-486-5p from human placental microvascular endothelial cells, on the function of trophoblast cells. To investigate the function of human placental microvascular endothelial cell (HPVEC)-derived exosomes on trophoblast cells, HPVECs were treated with hypoxia/reoxygenation (H/R). The separation efficiency of exosomes was determined by transmission electron microscopy, nanosight and Western blot. Cell Counting Kit-8, EdU staining, wound-healing, and transwell assay were performed to detect the effect of exosomally transferred miR-486-5p inhibitor on proliferation, migration and invasion of trophoblast cells. MiRDB and dual-luciferase report assay were used to find the target of miR-486-5p. Our data revealed that miR-486-5p was significantly upregulated in H/R-treated HPVEC-Exo, and miR-486-5p was enriched in HPVEC-Exo. miR-486-5p inhibitor carried by HPVEC-Exo significantly inhibited the proliferation, migration and invasion of trophoblast cells. Insulin-like growth factor 1 (IGF1) was found to be the target of miR-486-5p, and IGF1 overexpression notably reversed the effect of miR-486-5p inhibitor from HPVEC-Exo on trophoblast cell function. In summary, H/R-treated HPVEC-derived exosomally expressing miR-486-5p inhibitor significantly inhibited the proliferation, migration and invasion of trophoblast cells via downregulation of IGF1. The findings from the present study may be useful in the development of treatments for PE.

**Supplementary Information:**

The online version contains supplementary material available at 10.1007/s13577-021-00543-x.

## Introduction

Preeclampsia (PE) is a pregnancy-specific complication which manifests by blood pressure greater than 140/90 mmHg in the second half of pregnancy [1]. PE not only increases the risk of preterm birth and low birth weight babies, but is also a principal cause of maternal and fetal mortality [2]. Moreover, PE has long-term postpartum effects on the mother and the offspring later in life, such as increased risk of cardiovascular disease and hypertension [3]. Every year, 2–8% of all pregnant women suffer from PE globally [4].

Human placental microvascular endothelial cells (HPVECs) have been reported to play a crucial role in PE. For instance, downregulation of HIF-1a-mediated TLR4 activation may inhibit apoptosis and promote angiogenesis of HPVECs in PE [5]. In addition, pathogenetic implication of interleukin-2 expressed in preeclamptic decidual tissues: a possible mechanism of deranged vasculature of the placenta associated with PE [6]. On the other hand, trophoblast cells in placenta play an essential role in transport of nutrients and oxygen, secretion of pregnancy hormones, remodulation of the uterine arteries, and communication with maternal cells [7]. Although the exact pathophysiology of PE is not completely elucidated, it is broadly accepted that dysfunction of trophoblast cells is involved in the development and the progression of PE [8]. The dysregulation of trophoblast cells may impair the remodeling of the spiral arteries [9]. The impeded remodeling of the spiral arteries produces maternal endothelial cell dysfunction, contributes to poor placentation, and eventually results in clinical symptoms of PE [10]. Therefore, research on mechanisms of trophoblast cell and HPVEC dysregulation will be useful in revealing the etiology and pathophysiology of PE.

Exosomes are a type of extracellular vesicle (EV), with diameter ranging from 40 to 100 nm; exosomes are secreted by cells, and transfer signal molecule to targeted cells [11]. In addition, exosomes play key roles in the progression of multiple diseases [12, 13]. Previous reports indicating that exosomes can regulate the progression of PE [14] and have anti-inflammatory effects on trophoblast cells [15] suggest that exosomes are closely associated with the pathogenesis of PE. Meanwhile, there is evidence that exosomal has-miR-486-5p may regulate the progression of cancers and inflammation. For example, exosomal miR-486-5p from hypoxic tumor cells can promote the tumorigenesis of rectal cancer [16]. The PAX3-FOXO1 oncogene’s alteration of exosome miRNA content, which leads to paracrine effects, is mediated by exosomal miR-486-5p [17]. Transfer of miR-486-5p from human endothelial colony forming cell-derived exosomes has been shown to reverse ischemic kidney injury [18]. However, the function of exosomes derived from HPVECs expressing miR-486-5p in PE remains largely unknown.

Insulin-like growth factor 1 (IGF1) belongs to the IGF system, which is responsible for cell growth [19]. In addition, IGF1 is involved in multiple signaling pathways. For instance, IGF1 regulates PI3K signaling in heart failure [20]. In addition, IGF1 is regulated by insulin in rat testis and spermatogenic cells [21]. Meanwhile, IGF1 is targeted by miR-486-5p in hypertrophic scar fibroblasts [22]. Based on this previous research, we speculate that IGF1 might be regulated by exosomal miR-486-5p in PE. The findings from those studies suggest that HPVECs may be a key mediator in PE via exosomal miR-486-5p. Thus, in this study, we investigated the effect of exosomes derived from HPVECs expressing miR-486-5p on progression of PE in vitro, with the goal of gaining additional insights for the treatment of PE.

## Materials and methods

### Cell culture and treatment

Human placental microvascular endothelial cells (HPVECs), HTR8/SVneo cells and TEV1 cells were purchased from the Sciencell (Shanghai, China) and cultured in Dulbecco’s Modified Eagle’s Medium (DMEM, Thermo Fisher Scientific, Waltham, MA, USA), supplemented with 10% fetal bovine serum (FBS), 1% penicillin and streptomycin (Thermo Fisher Scientific) in an incubator (37 °C, 5% CO_2_). The PE model was established in vitro. HPVECs were placed in deoxygenated medium and then onto the hypoxic vessel, supplemented with a mixture of 95% N_2_ and 5% CO_2_ for 4 h at 37 °C after cell transfection. The HPVECs were transferred to DMEM supplemented with 10% FBS under normoxic conditions (5% CO_2_) at 37 °C for 24 h. TEV1 cells originated from first-trimester normal extravillous cytotrophoblasts, which were isolated from human placenta by human papillomavirus pLXSN-E6/E7 open reading frames transfection, as previously described [23].

### Cell transfection

siRNA targeted against HIF-1a (HIF-1a siRNA; 10 nM) and a negative control siRNA (siRNA-NC) were purchased from Guangzhou RiboBio Co., Ltd. and transfected into HPVECs (5 × 10^3^) using Lipofectamine^®^ 2000 (Thermo Fisher Scientific, Inc.), according to the manufacturer’s instructions. Cells were incubated at 37 °C for 6 h before subsequent experiments were performed.

### Exosome isolation

HPVECs were plated in a 10 cm dish at a density of 1 × 10^6^ cells per dish with DMEM. The culture medium was discarded after 72 h, the cells were washed 3 times in serum-free medium, and 10 ml serum-free medium was added to each dish. After 48 h, HPVECs were centrifuged twice at 2000 rpm per minute, and the HIEFF™ Quick exosome isolation kit (41201ES50, Yeasen, Shanghai, China) was used to extract exosomes. Then, the cell supernatant and isolated exosomes (2:1) were added into the centrifuge tube for incubation overnight at 4 °C. On the following day, the mixture was centrifuged at 10,000 rpm per minute at 4 °C for 1–2 h. The supernatant was removed, whereas the precipitate (exosomes) was collected. Based on the volume ratio of the initial medium and the resuspension (10:1), the precipitate was resuspended in phosphate-buffered saline (PBS). Then, 30 μL of the resuspension (exosomes) was placed in an Eppendorf tube and mixed with the radio-Immunoprecipitation Assay (RIPA) lysis buffer of equal volume and maintained on ice. Microwave methods were employed to lyse the mixture twice, and the bicinchoninic acid (BCA) protein assay kit (Beyotime Biotechnology, Jiangsu, China) was used to determine the protein concentration in exosomes.

### Transmission electron microscopy

Exosome pellets were placed on a carbon-coated copper grid and incubated for 5 min at 37 °C and then immersed in 2% phosphotungstic acid solution for 1 min. After washing with PBS, the preparations were captured using transmission electron microscopy (TEM) (JEOL, Akishima, Japan).

### Nanosight tracking analysis

Nanosight Tracking Analysis (NTA) (NanoSight NS300, Malvern Instruments, UK) was used for size distribution and concentration measurements of exosomes in liquid suspension from the properties of both light scattering and Brownian motion. The NanoSight NS300 with a 405 nm laser instrument was used to detect nanovesicles. For each sample, five video clips, each 60 s long, were taken. Data were analyzed using the NTA 3.0 software, and hydrodynamic diameters of each particle were calculated using the Stokes–Einstein equation: *D* = *kT/6πηr*, where *D* is the diffusion coefficient, *k* is Boltzmann’s constant, and *T* is the absolute temperature, *r* is the radius of the particle, and *η* is the viscosity of the fluid, based on a spherical particle moving with the uniform velocity in a continuous fluid.

### HTR8/SVneo and TEV1 co-cultured with HPVEC-exo

After the exosomes were isolated from HPVECs, subsequent procedures were conducted. Briefly, the exosomes were resuspended in 1 mL diluent solution. Next, 1 mL of exosome suspension was mixed with the dye solution for 5 min. After adding 1.5 mL of sucrose solution to the exosomes, the samples were centrifuged at 100,000 rpm per minute for 2 h at 2–8 °C. The exosomal pellets were resuspended in PBS and transferred to an Amicon filter column. After adding 9 mL of PBS and 0.75 mL of medium, the exosomes were then centrifuged at high speeds (2000 per minute) for 40 min to reduce the volume to 0.5–1 mL.

HTR8/SVneo and TEV1 cells were routinely cultured and seeded, and the medium was renewed after 48 h. Cells were then incubated with exosomes or PBS for 24 h. Then, cells were then treated with PBS or co-cultured with exosomes isolated from HPVECs, from negative control (NC)-transfected HPVECs or from miR-486-5p inhibitor-transfected HPVECs.

### RNA transfection

For RNA transfection, HPVECs were transfected with miR-486-5p inhibitor for 24 h using Lipofectamine^®^ 2000 (Thermo Fisher Scientific, Inc.), according to the manufacturer’s instructions.

### Vector construction and IGF1 overexpression

RNA interference target sequences were designed using the IGF1 gene as a template to construct a target gene RNA interference pcDNA3.1 vector (Shanghai Biosciences, Co., Ltd., Shanghai, China). The single-stranded DNA oligo containing interference sequence was synthesized, and the obtained lyophilized powder was dissolved in an annealing buffer (final concentration, 100 M) and incubated in a water bath at 90 °C for 15 min. After naturally cooling to room temperature, a double-stranded oligo with overhang ends was formed, which was then directly ligated into the digested lentiviral vector through restriction sites at both ends. The 50-μl reaction system was prepared according to instructions from New England Biolabs, Inc. The BR-V-108 vector was linearized by double digestion, and the ligation product was introduced into prepared TOP10 coli competent cells. The sequencing results were compared with those of the correct clones, and the plasmid was isolated. For cell transfection, TEV1 or HTR8/SVneo cells were transfected with the pcDNA3.1 vector (NC) or pcDNA3.1-IGF1 (IGF1 overexpression) using Lipofectamine 3000 (Invitrogen, Carlsbad, CA, USA). The pcDNA3.1 vector and pcDNA3.1-IGF1 were purchased from GenePharma (Shanghai, China). The supernatant was collected after 48 h of incubation. After HTR8/SVneo and TEV1 cells were transfected with pcDNA3.1-IGF1, they were co-cultured with (hypoxia/reoxygenation [H/R] + NC inhibitor)-exo or (H/R + miR-486-5p inhibitor)-exo.

### Dual-luciferase reporter assay

IGF1 was found to be the target of miR-486-5p according to the MicroRNA Target Prediction Database tool, and IGF1 may regulate the migration of trophoblast cells as previously described [24]. Thus, dual-luciferase reporter assay was performed to confirm the correlation between miR-486-5p and IGF1. 3′-UTR of IGF1 containing the putative binding sites of miR-486-5p were synthetized and obtained from Sangon Biotech (Shanghai, China), and were then cloned into the pmirGLO Dual-Luciferase miRNA Target Expression Vectors (Promega, Madison, WI, USA) to construct wild-type reporter vectors IGF1 (WT), respectively. The IGF1 (WT) or IGF1 (MUT) was transfected into 293T cells using Lipofectamine 2000 (Thermo Fisher Scientific) according to the manufacturer’s instructions. The relative luciferase activity was analyzed by the Dual-Glo Luciferase Assay System (Promega).

### Quantitative real-time polymerase chain reaction (RT-qPCR)

Total RNA from cells was extracted using TRIzol reagent (Invitrogen, Carlsbad, CA, USA), and total RNA from exosomes was isolated with the exoRNeasy Midi Kit (Qiagen, Valencia, CA, USA) following the standard procedure. Then, total RNA was reversely transcribed into complementary DNA (cDNA) using All-in-One™ Kit (FulenGen, Guangzhou, China). After that, RT-qPCR was performed by SYBR™ Green Master Mix (Qiagen). Relative transcription alterations were analyzed by 2^−ΔΔCt^ method and normalized by GADPH. The specific primer sequences were listed as follows: IGF1: F, 5′-ATCTAATCAGTAAGCGGA-3′ and R, 5′-AAAGCAAGTCAAGACCTC-3′; miR-486-5p: F, 5′-GAAAAATTGAACCACCCGGCA-3′ and R, 5′-TTCCAAGGAGTTGCTCCCGT-3′, U6: F, 5′-GAAAAATTGAACCACCCGGCA-3′ and R, 5′-TTCCAAGGAGTTGCTCCCGT-3′; GADPH: F 5′-ACAGCAACAGGGTGGTGGAC-3′ and R 5′-TTTGAGGGTGCAGCGAACTT-3’.

### CCK-8 assay

Cell counting kit-8 assay (CCK-8; Beyotime Institute of Biotechnology) was used to investigate cell proliferation. Cells were plated into 96-well plates at a density of 5 × 10^3^ cells per well. After treatment, cells were then incubated with 10 μl CCK-8 reagent for another 2 h at 37 °C. Subsequently, the absorbance of cells was measured at 450 nm using a microplate reader (Thermo Fisher Scientific, Inc.).

### Western blot

Total proteins were lysed using RIPA lysis buffer. The concentration of protein was then detected using a BCA protein kit (Thermo Fisher Scientific, Inc.). Proteins (40 μg per lane) were separated via 10% sodium dodecyl sulfate–polyacrylamide gel electrophoresis, and then transferred onto polyvinylidene difluoride membranes (Thermo Fisher Scientific, Inc.). Subsequently, the membranes were blocked with 5% skim milk in Tris-buffered saline with 0.1% Tween ^®^ 20 detergent for 1 h at room temperature, and then incubated with the primary antibodies against IGF1 (Abcam, 1:1,000) or β-actin (Abcam, 1:1,000) overnight at 4 °C. The membranes were then incubated with horse radish peroxidase-conjugated secondary antibodies for 1 h at room temperature. Finally, the protein bands were detected using Enhanced Chemiluminescence kit (Thermo Fisher Scientific, Inc.). β-Actin was used as an internal control. Image-Pro Plus 6.0 (Media Cybernetics, Inc.) was used for densitometry analysis.

### EdU staining

Cells were seeded onto 24-well plates overnight (5 × 10^4^ cells per well). Cells were prefixed in 4% paraformaldehyde for 10 min, and fixed in pre-cold methanol for another 10 min. Subsequently, cells were incubated with the following primary antibodies overnight at 4 °C: anti-5-ethynyl-2′-deoxyuridine (EdU) (Abcam; 1:1,000), and nuclei were stained with 4′,6-diamidino-2-phenylindole (Genepharma). Goat anti-rabbit IgG antibody (Abcam; 1:5,000) was used as the secondary antibody. The samples were visualized using a fluorescence microscope (CX23, Olympus Corporation) immediately after staining.

### Transwell assay

The upper chamber was pre-treated with 100 μl of Matrigel. Cells were seeded onto the upper chamber in media with 1% FBS, and the density was adjusted to about 1 × 10^6^ cells per chamber. RPMI1640 medium with 10% FBS was added to the lower chamber. After 24 h of incubation at 37 °C, the transwell chamber was rinsed twice with PBS (5 min each time), fixed by 5% glutaraldehyde at 4 °C and stained with 0.1% crystal violet for 30 min. The transwell chamber was washed twice with PBS and then observed under a microscope. The number of cells invading the Matrigel was regarded to be a reflection of the invasion ability.

### Wound-healing assay

Cells were plated onto a 24-well cell culture cluster, and were allowed to grow to 80–90% confluence. Then, the wells were scratched with a small pipette tip. After washing with PBS 3 times, serum-free medium was used for further culture, and the scratch widths at 0 and 48 h were recorded under an optical microscope. The experiment was repeated 3 times.

### Statistical analysis

Three independent experiments were performed for each group, and all data were expressed as the mean ± standard deviation (SD). Differences were analyzed using Student’s *t *test (only 2 groups) or one-way analysis of variance (ANOVA) followed by Tukey’s test if more than 2 groups were compared, (GraphPad Prism7). *P* < 0.05 was considered to indicate a statistically significant difference.

## Results

### Exosomes were successfully separated from HPVECs

**Fig. 1:**
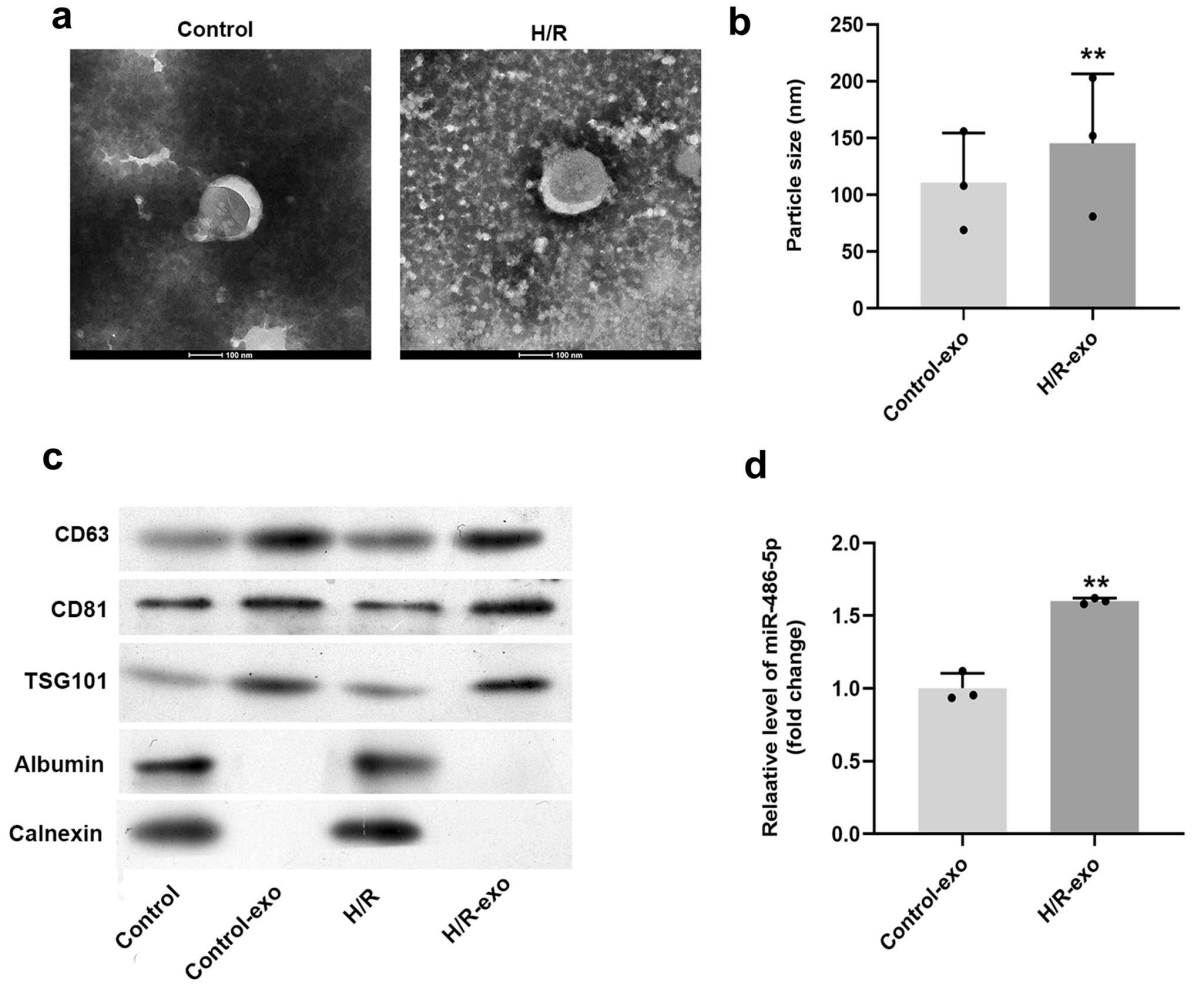
Exosomes were successfully separated from HPVECs. Exosomes were isolated from HPVECs. **a** Extracellular vesicles showed disc-shaped, crescent-shaped, and double-layered membrane structure, and rounded particles typically ranging from 30 to 150 nm in diameter were observed. **b** Compared with the control group, the particle sizes of extracellular vesicles isolated from H/R-treated cells were significantly increased. **c** The expression levels of CD63, CD81 and TSG101 in extracellular vesicles were notably higher than those in control or H/R-treated cells. **d** The level of miR-486-5p in extracellular vesicles derived from H/R-treated HPVECs was significantly higher, compared with control. ^**^P < 0.01 compared to control, *n* = 3

The separation efficiency of exosomes was examined by TEM. As revealed in Fig. 1a, exosomes had a disc-shaped, crescent-shaped, and double-layered membrane structure, and rounded particles typically ranged from 30 to 150 nm in diameter. Compared with the control group, the particle sizes of exosomes isolated from H/R-treated cells were significantly increased (Fig. 1b). The expression levels of CD63, CD81 and TSG101 in exosomes were notably higher than those in control or H/R-treated cells (Fig. 1c). In contrast, calnexin and albumin were rarely expressed in exosomes (Fig. 1c). The level of miR-486-5p in exosomes derived from H/R-treated HPVECs was significantly higher, compared with control (Fig. 1d). Based on these data, these features were consistent with exosomes. Thus, exosomes were successfully separated from HPVECs.

### Exosomes derived from H/R-induced HPVECs significantly inhibited the proliferation of trophoblast cells

**Fig. 2:**
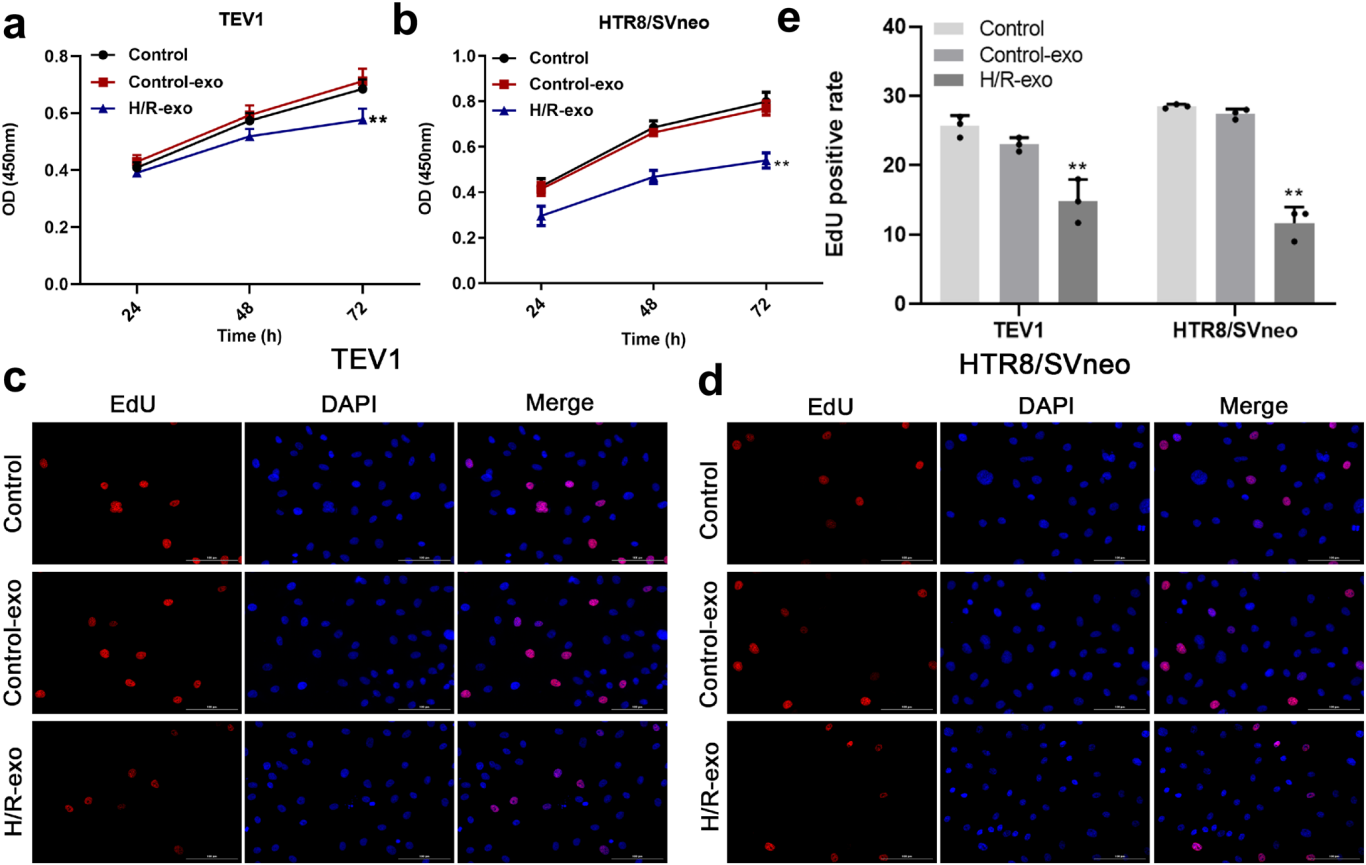
Exosomes derived from H/R-induced HPVECs significantly inhibited the proliferation of trophoblast cells. **a**, **b** Exosomes derived from H/R-induced HPVECs significantly decreased the viability of **a** TEV1 and **b** HTR8/SVneo cells. The positive rate of EdU staining in **c** TEV1 and **d** HTR8/SVneo cells was also inhibited in the presence of exosomes derived from H/R-induced HPVECs. **e** The positive rate of EdU staining was calculated. ^**^P < 0.01 compared to control, *n* = 3

To investigate the effect of exosomes on proliferation of trophoblast cells, CCK-8 assay was performed. The data revealed that exosomes derived from H/R-induced HPVECs significantly decreased the viability of TEV1 or HTR8/SVNEO cells (Fig. 2a, b), and the positive rate of EdU staining in trophoblast cells was also decreased in the presence of exosomes derived from H/R-induced HPVECs (Fig. 2c–e). In summary, exosomes derived from H/R-induced HPVECs significantly inhibited the proliferation of trophoblast cells.

### Exosomes derived from H/R-induced HPVECs significantly decreased the migration and invasion of trophoblast cells

**Fig. 3:**
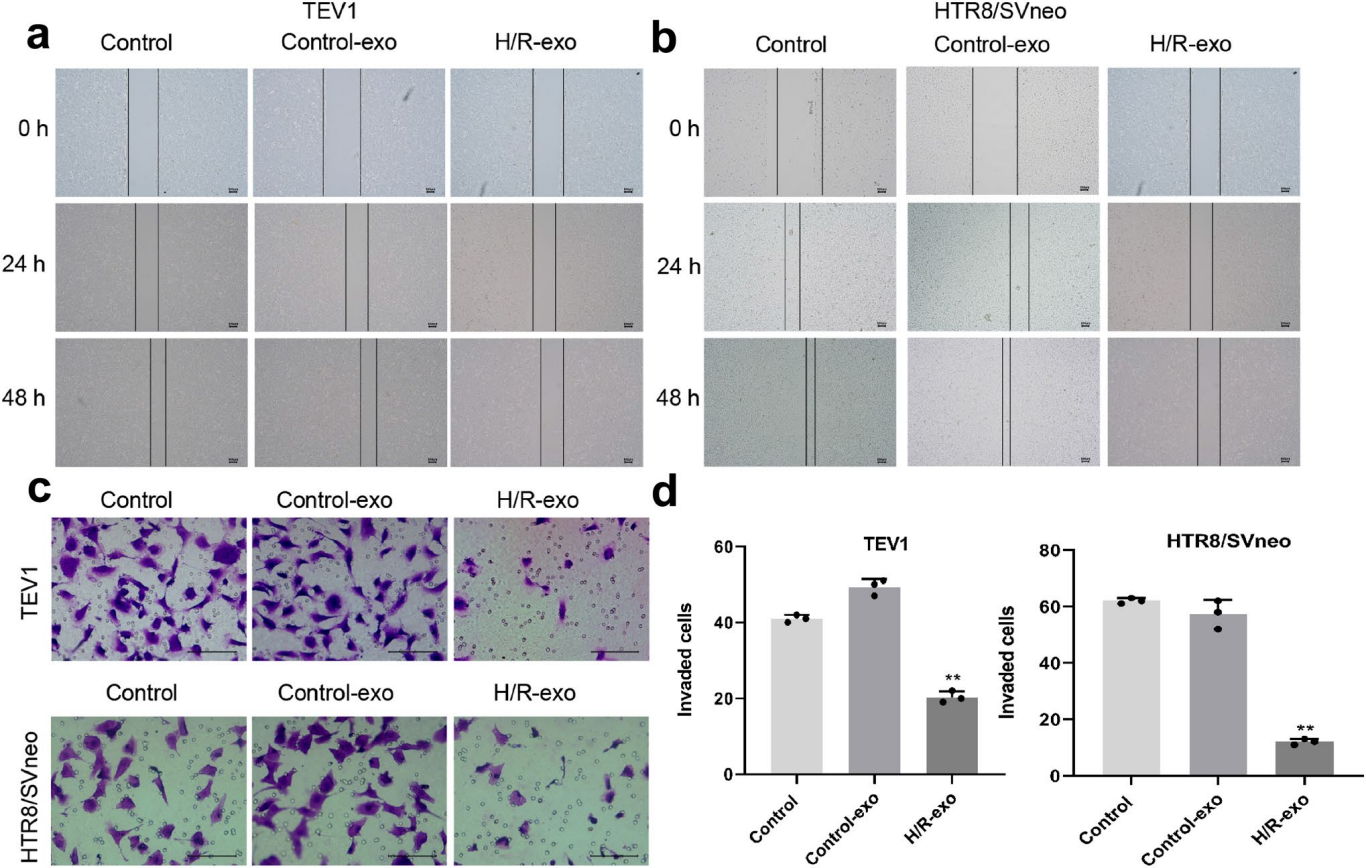
Exosomes derived from H/R-induced HPVECs significantly decreased the migration and invasion of trophoblast cells. The migration of **a** TEV1 or **b** HTR8/SVNEO cells was notably decreased by exosomes derived from H/R-induced HPVECs. **c**, **d** The invasion of TEV1 or HTR8/SVNEO cells was notably decreased by exosomes derived from H/R-induced HPVECs. **e** The invaded cells were counted under a microscope. ^**^P < 0.01 compared to control, *n* = 3

To detect the effect of exosomes derived from H/R-induced HPVECs on migration of trophoblast cells, wound-healing assays were performed. As shown in Fig. 3a, b, the migration of TEV1 or HTR8/SVNEO cells was notably decreased by exosomes derived from H/R-induced HPVECs, and the transwell assay suggested that exosomes derived from H/R-induced HPVECs decreased the invasion of trophoblast cells (Fig. 3c, d). To sum up, exosomes derived from H/R-induced HPVECs significantly decreased the migration and invasion of trophoblast cells.

### Exosomes derived from H/R-treated HPVECs delivered miR-486-5p to trophoblast cells

**Fig. 4:**
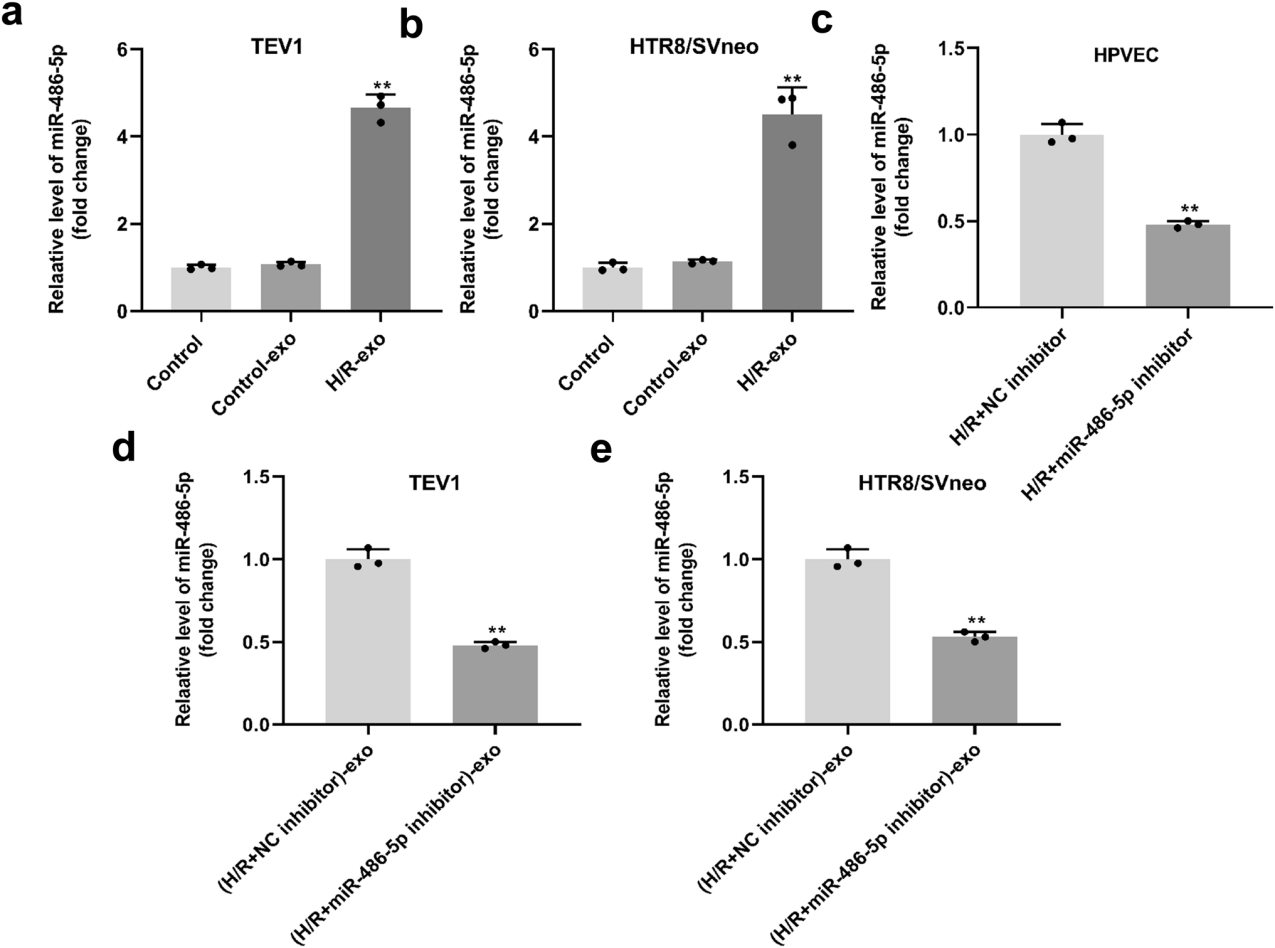
Exosomes derived from H/R-treated HPVECs deliver miR-486-5p to trophoblast cells. The expression of miR-486-5p in **a** TEV1 or **b** HTR8/SVneo cells was significantly increased by exosomes derived from H/R-treated HPVECs. **c** miR-486-5p expression in H/R-treated HPVECs was downregulated by miR-486-5p inhibitor. The level of miR-486-5p was downregulated by exosomes derived from H/R-treated HPVECs with downregulated miR-486-5p in **d** TEV1 or **e** HTR8/SVneo cells. ^**^P < 0.01 compared to control, *n* = 3

To detect miR-486-5p expression, RT-qPCR was used. As indicated in Fig. 4a, b, the expression of miR-486-5p in trophoblast cells was significantly increased by exosomes derived from H/R-treated HPVECs. MiR-486-5p expression in HPVECs was downregulated by miR-486-5p inhibitor (Fig. 4c), and the level of miR-486-5p in trophoblast cells was downregulated by exosomes derived from H/R-treated HPVECs with downregulated miR-486-5p (Fig. 4d, e). In summary, exosomes derived from H/R-treated HPVECs delivered miR-486-5p to trophoblast cells.

### Exosomes derived from HPVECs delivered miR-486-5p to inhibit the proliferation of trophoblast cells

**Fig. 5:**
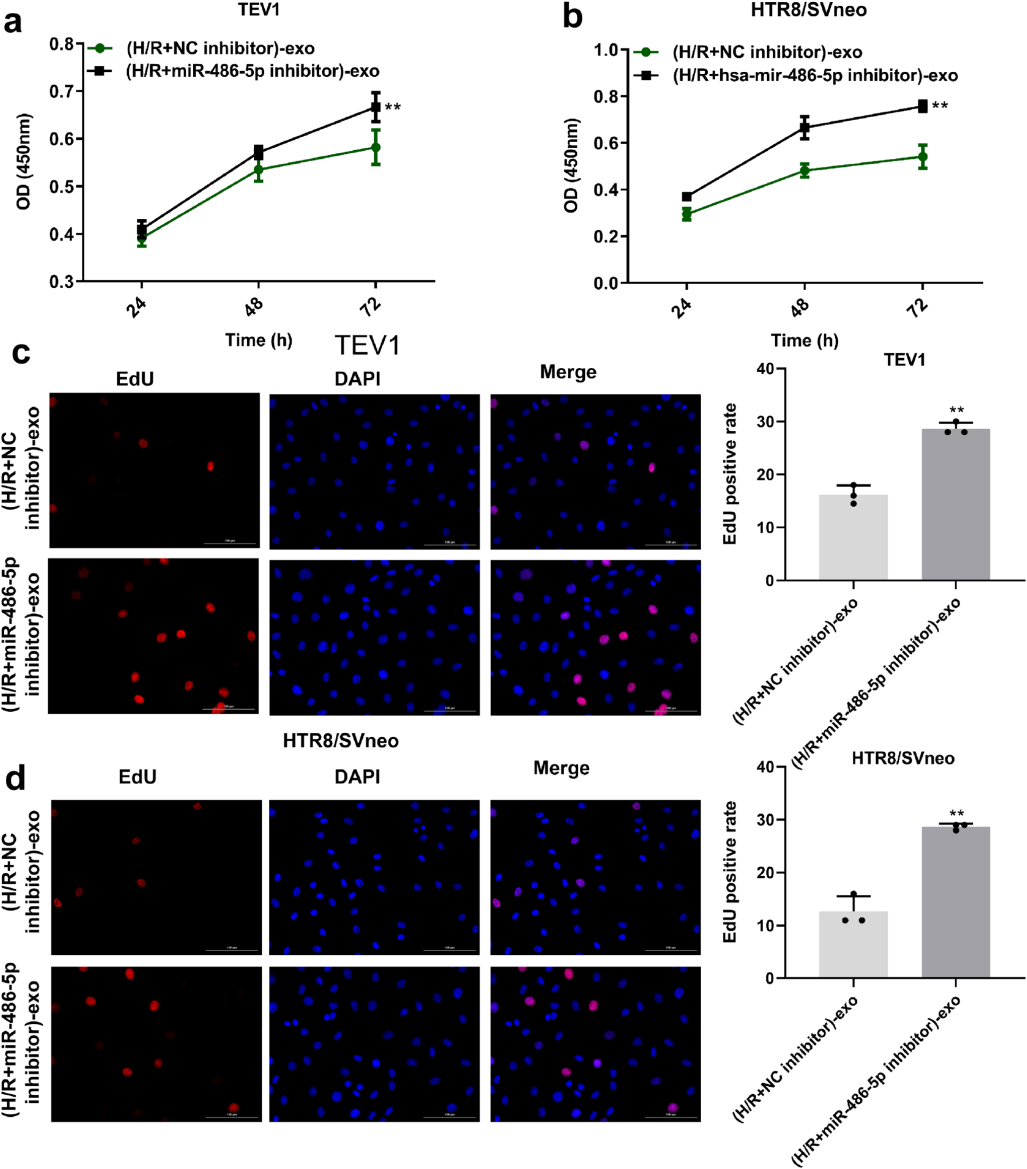
Exosomes derived from HPVECs deliver miR-486-5p to inhibit the proliferation of trophoblast cells. miR-486-5p was significantly upregulated in H/R-treated HPVEC-exo, and miR-486-5p inhibitor carried by HPVEC-exo significantly inhibited the viability of **a** TEV1 and **b** HTR8/SVneo cells. MiR-486-5p inhibitor carried by HPVEC-exo significantly suppressed the EdU positive rate in **c** TEV1 and **d** HTR8/SVneo cells. ^**^P < 0.01 compared to (H/R + NC inhibitor)-exo, *n* = 3

To detect the function of exosomal miR-486-5p in trophoblast cells, CCK-8 and EdU staining were used. The data showed that miR-486-5p was significantly upregulated in H/R-treated HPVEC-exo and that miR-486-5p inhibitor carried by HPVEC-exo significantly inhibited the viability and proliferation of trophoblast cells (Fig. 5a–d). Taken together, the findings suggest that miR-486-5p from HPVEC-derived exosomes inhibited the proliferation of trophoblast cells.

### Exosomes derived from HPVECs delivered miR-486-5p to decrease the migration and invasion of trophoblast cells

**Fig. 6:**
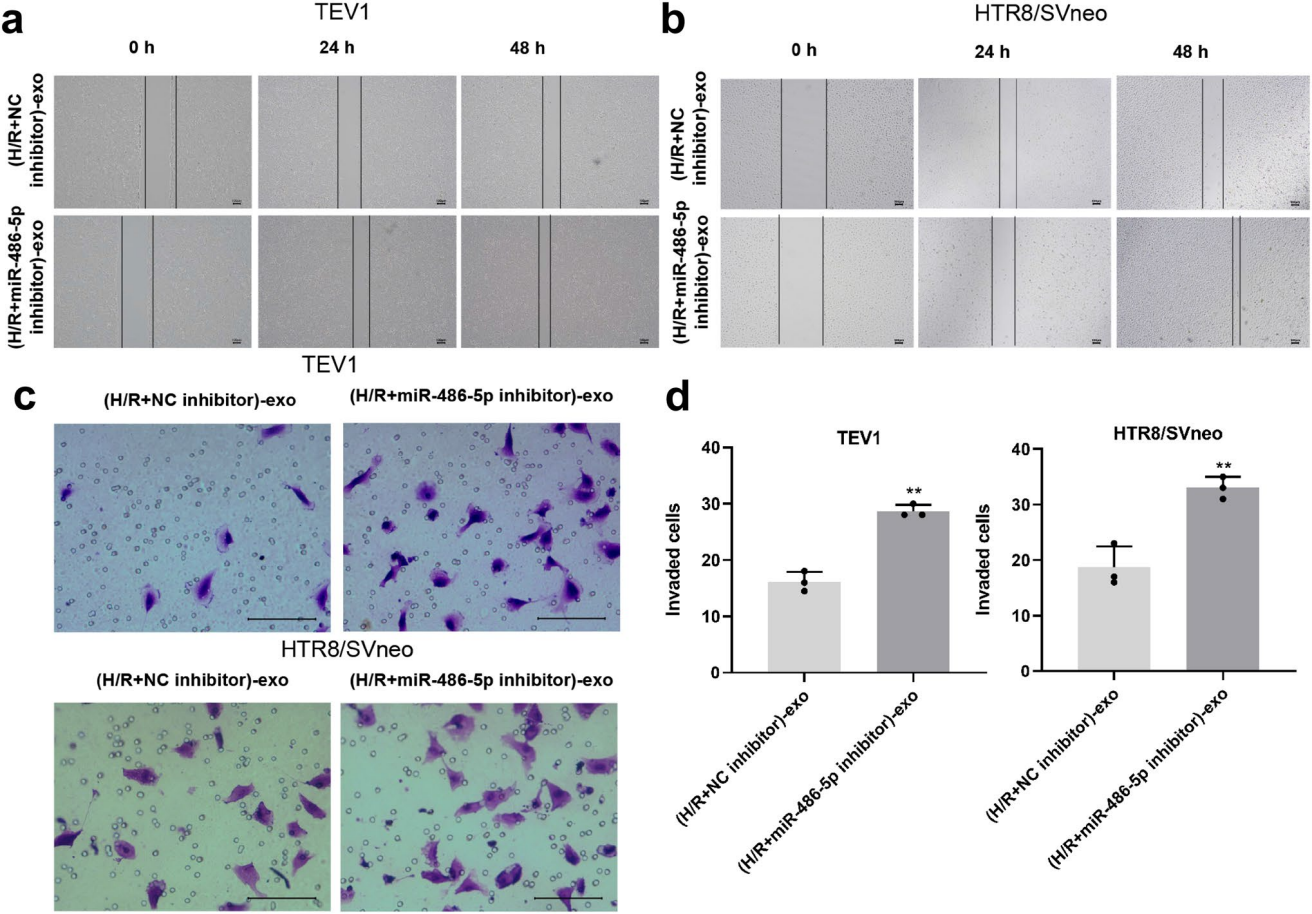
Exosomes derived from HPVECs deliver miR-486-5p to inhibit the migration and invasion of trophoblast cells. MiR-486-5p inhibitor carried by HPVEC-exo significantly inhibited the migration of **a** TEV1 and **b** HTR8/SVneo cells. **c** The invasion of TEV1 and HTR8/SVneo cells was significantly decreased in the presence of (H-R + miR-486-5p inhibitor)-exo. **d** The invaded cells were counted under a microscope. ^**^P < 0.01 compared to compared to (H/R + NC inhibitor)-exo, *n* = 3

For the purpose of investigating the effect of exosomes on migration of trophoblast cells, wound-healing assay was used. As revealed in Fig. 6a, b, miR-486-5p inhibitor carried by HPVEC-exo significantly inhibited the migration of trophoblast cells. Consistent with this finding, the invasion of trophoblast cells was significantly decreased in the presence of (H/R + miR-486-5p inhibitor)-exo (Fig. 6c, d). Altogether these results suggest that miR-486-5p from HPVEC-derived exosomes decreased the migration and invasion of trophoblast cells.

### MiR-486-5p directly targeted IGF1 in trophoblast cells

**Fig. 7:**
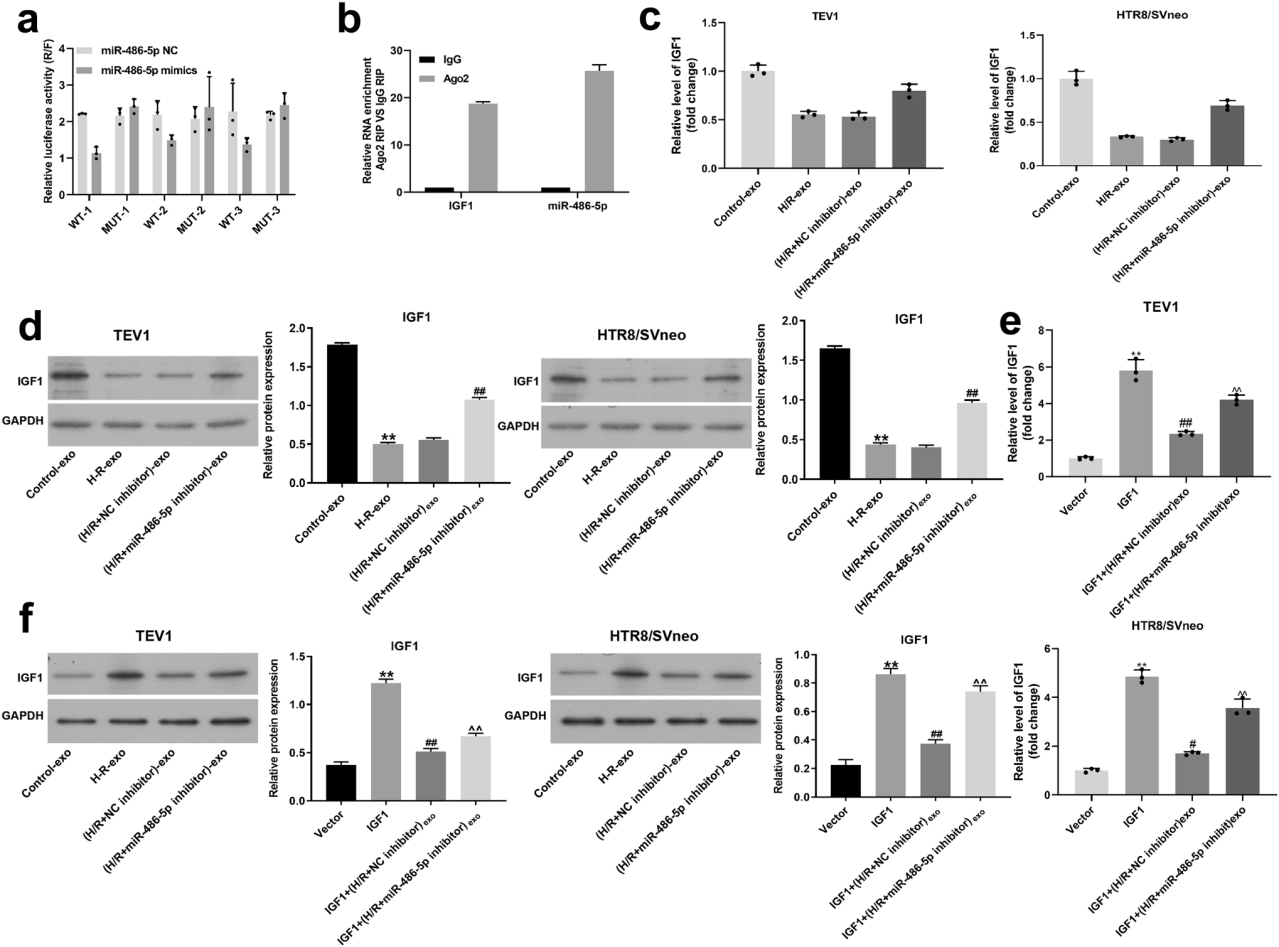
miR-486-5p directly targeted IGF1 in trophoblast cells.** a** The relative luciferase activity in WT-IGF1 was significantly decreased by miR-486-5p mimics. **b** The enrichment of IGF1 and miR-486-5p in trophoblast cells was significantly upregulated by Ago2. **c** The expression of IGF1 in trophoblast cells was notably upregulated when transfected with IGF1 overexpression, while this phenomenon was partially reversed by (H/R + miR-486-5p inhibitor)-exo. **d** miR-486-5p inhibitor carried by HPVEC-exo partially rescued the effect of H/R-exo on IGF1 expression. **e**,** f** miR-486-5p inhibitor carried by HPVEC-exo partially rescued the effect of IGF1 overexpression on IGF1 expression. ^**^P < 0.01 compared to control. ^##^*P* < 0.01 compared to H/R-exo. ^^^^*P* < 0.01 compared to (H/R + NC inhibitor)-exo, *n* = 3

Given that IGF1 was found to be the target of niR-486-5p, the correlation was further investigated by dual-luciferase report assay. The result suggested that relative luciferase activity in WT-IGF1 was significantly decreased by miR-486-5p mimics (Fig. 7a) and the binding sites are shown in Table 1. However, the enrichment of IGF1 in trophoblast cells was significantly upregulated by miR-486-5p mimics (Fig. 7b) Compared with control, the expression of IGF1 in trophoblast cells was notably downregulated when treated with H/R-exo, while this phenomenon was partially reversed by (H/R + miR-486-5p inhibitor)-exo (Fig. 7c, d). Meanwhile, miR-486-5p inhibitor carried by HPVEC-exo partially rescued the effect of IGF1 overexpression on IGF1 expression (Fig. 7e, f). In summary, miR-486-5p directly targeted IGF1 in trophoblast cells.

**Table 1:** The binding sites between miR-486-5p and IGF1 are shown

Binding site	Predicted consequential pairing of target region (top) and miRNA (bottom)
IGF1-1 3′ UTR miR-486-5p	5′-CUUUAAGUGCAUAUGGUACAGGA-3′ 3′-GAGCCCCGUCGAGUCAUGUCCU-5′
IGF1-2 3′ UTR miR-486-5p	5′-UUAGCAUAUCAAUUAUACAGGAU-3′ 3′-GAGCCCCGUCGAGUCAUGUCCU-5′
IGF1-3 3′ UTR miR-486-5p	5′-GCAGGAAACAAGAACUACAGGAU-3′ 3′-GAGCCCCGUCGAGUCAUGUCCU-5′

### Exosomes derived from HPVECs delivered miR-486-5p to inhibit the proliferation of trophoblast cells via targeting IGF1

**Fig. 8:**
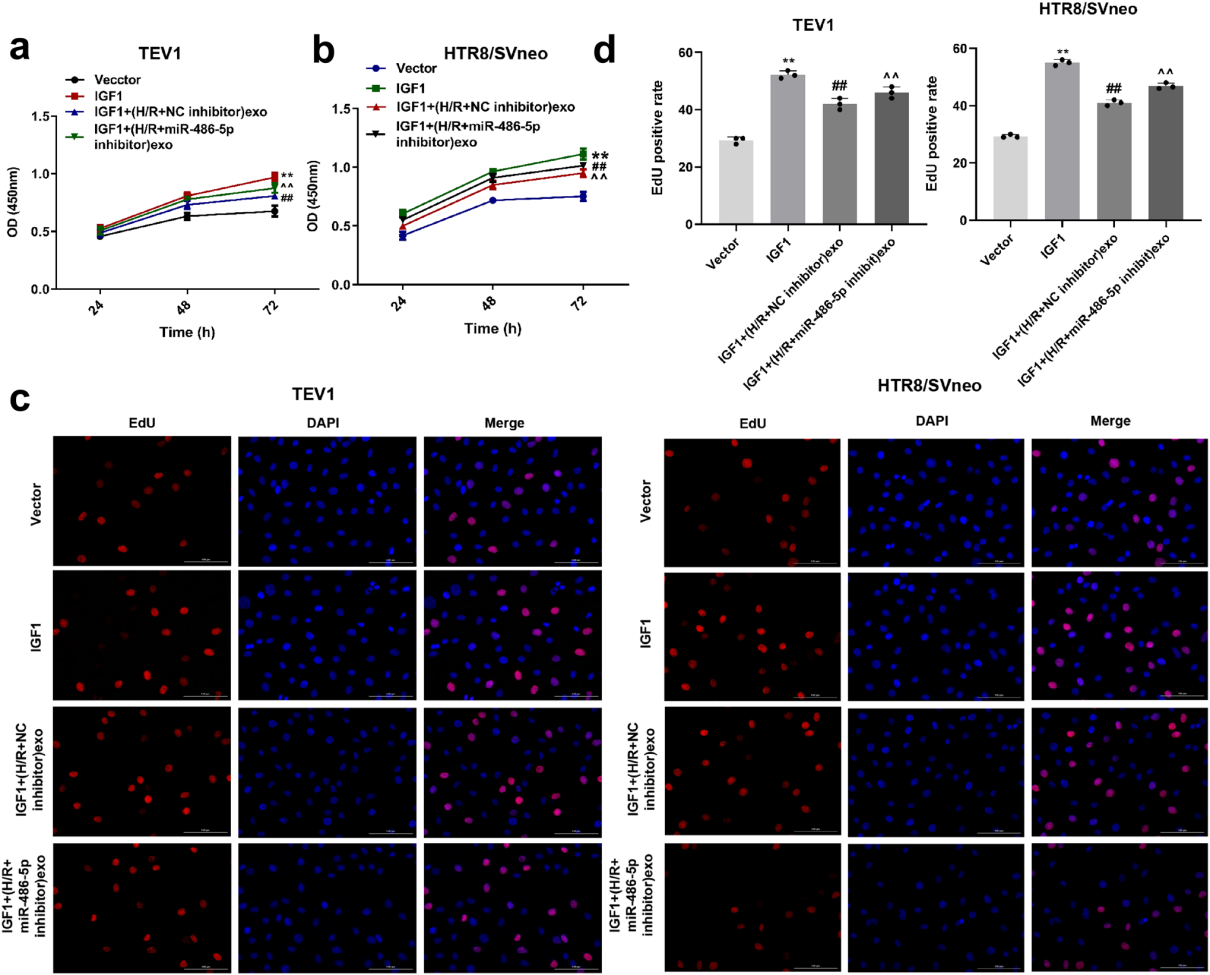
Exosomes derived from HPVECs deliver miR-486-5p to inhibit the proliferation of trophoblast cells via targeting IGF1. IGF1 overexpression significantly increased the viability of **a** TEV1 and **b** HTR8/SVneo cells, while (H/R + miR-486-5p inhibitor)-exo reversed this phenomenon. **c** IGF1 overexpression significantly increased the proliferation of TEV1 and HTR8/SVneo cells, while (H/R + miR-486-5p inhibitor)-exo reversed this phenomenon. **d** The positive rate of EdU staining was counted. ^**^P < 0.01 compared to control. ^##^*P* < 0.01 compared to H/R-exo. ^^^^*P* < 0.01 compared to (H/R + NC inhibitor)-exo, *n* = 3

To detect the role of IGF1 in PE, the CCK-8 assay was used. The data revealed that IGF1 overexpression significantly increased the viability and proliferation of trophoblast cells, while (H/R + miR-486-5p inhibitor)-exo reversed this phenomenon (Fig. 8a–d). Taken together, the data suggest that miR-486-5p from HPVEC-derived exosomes inhibited the proliferation of trophoblast cells via targeting IGF1.

### Exosomes derived from HPVECs delivered miR-486-5p to inhibit the migration and invasion of trophoblast cells via targeting IGF1

**Fig. 9:**
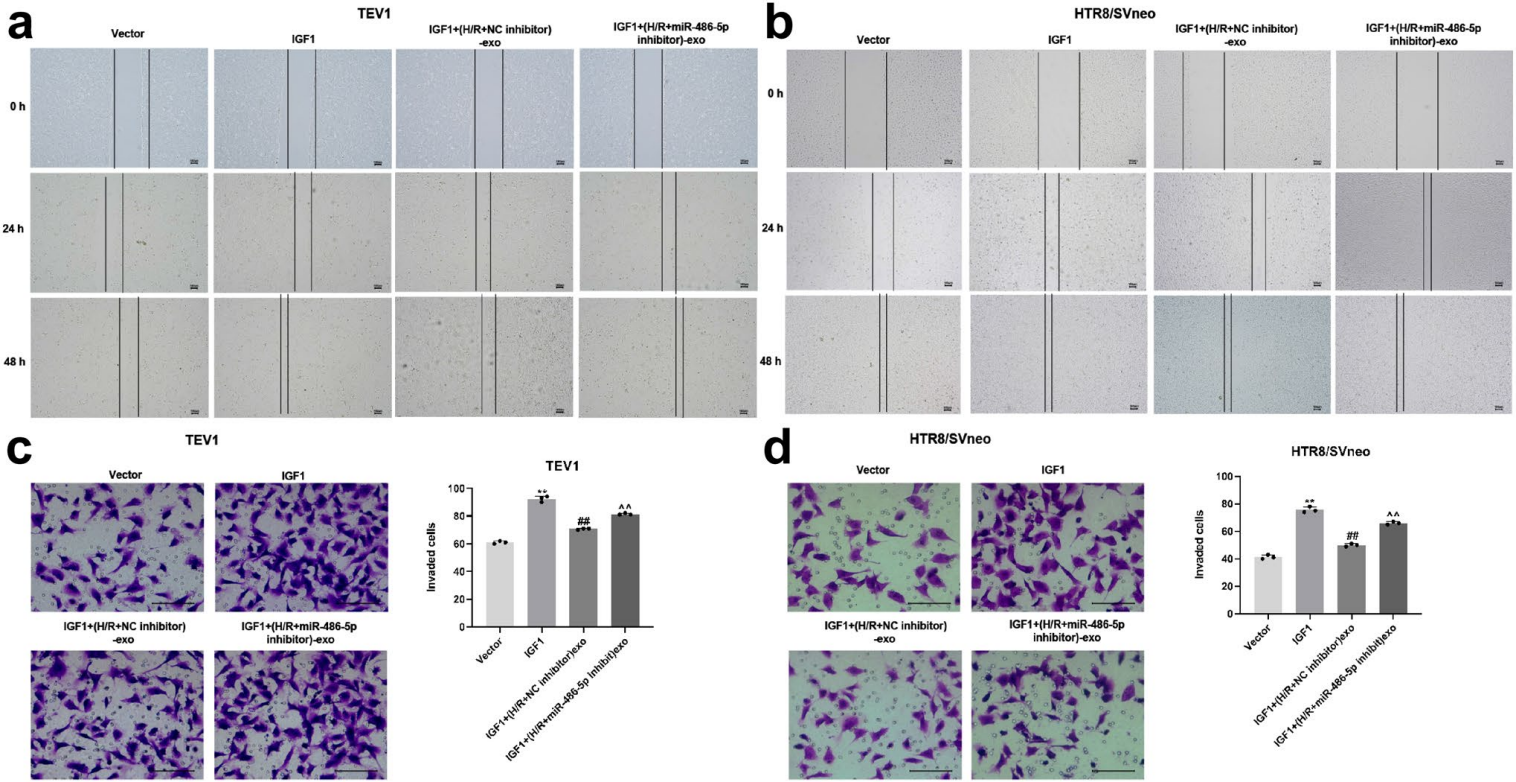
Exosomes derived from HPVECs deliver miR-486-5p to inhibit the migration and invasion of trophoblast cells via targeting IGF1. IGF1 overexpression significantly increased the migration of **a** TEV1 and **b** HTR8/SVneo cells, while (H/R + miR-486-5p inhibitor)-exo reversed the effect of IGF1 overexpression. IGF1 overexpression significantly increased the invasion of **c** TEV1 and **d** HTR8/SVneo cells, while (H/R + miR-486-5p inhibitor)-exo reversed the effect of IGF1 overexpression. ^**^P < 0.01 compared to control. ^##^*P* < 0.01 compared to H/R-exo. ^^^^*P* < 0.01 compared to (H/R + NC inhibitor)-exo

To detect the cell migration and invasion, wound-healing and transwell assays were performed. The data revealed that IGF1 overexpression significantly increased the migration and invasion of trophoblast cells, while (H/R + miR-486-5p inhibitor)-exo reversed the effect of IGF1 overexpression (Fig. 9a–d). These data suggest that miR-486-5p from HPVEC-derived exosomes inhibited the migration and invasion of trophoblast cells via targeting IGF1.

## Discussion

Previous research has confirmed that exosomes play a role in multiple conditions, including PE [11, 25, 26]. Furthermore, trophoblast cell growth inhibition, and dysregulation of miRNAs are involved in the progression of PE [27, 28]. In the current study, we found that the growth of trophoblast cells was significantly inhibited by miR-486-5p inhibitor carried by HPVEC-exo. The current study also revealed a possible mechanism by which exosomes modulate the progression of PE, by showing that miR-486-5p inhibitor carried by HPVEC-exo may serve as a key mediator in PE. A recent study found that exosomal miRNA-486-5p derived from rheumatoid arthritis fibroblast-like synoviocytes may induce osteoblast differentiation through the Tob1/BMP/Smad pathway [29]. The present study found that exosomes derived from H/R-treated HPVECs may deliver miR-486-5p to trophoblast cells. Thus, additional functions of exosomal miR-486-5p need to be investigated.

This study confirmed that IGF1 was the direct target of miR-486-5p. IGF1 has been confirmed to act as a mediator in some diseases [30–32]. Previous research found that IGF1 overexpression may promote the migration of trophoblast cells [24]. In this study, the effect of IGF1 on the progression of PE was investigated. As expected, our current finding was consistent with the previous report, which showed that, IGF1 was targeted by miR-30a-3p. Additional miRNAs may, therefore, be involved in the progression of PE, and the relationship between miRNAs and exosomes should be further explored. MiR-486-5p may target other mRNAs besides IGF1. For instance, YAP1 is targeted by miR-486-5p in ovarian cancer cells [33]. In addition, miR-486-5p may mediate the growth of gastric cancer cells via targeting of PI3KR1 [34]. Therefore, additional mRNAs targets of miR-486-5p in PE need further investigation.

The limitations of the present research should be considered. The mechanism by which IGF1 mediates the progression of PE needs to be explored in depth, and the identity of miRNAs involved in PE should be identified.

In summary, exosomes derived from human placental microvascular endothelial cells deliver miR-486-5p, which induces PE via targeting of IGF1. Our data provide additional insights that will hopefully advance the search for treatments for PE.

## Supplementary Information

Below is the link to the electronic supplementary material.

Supplementary file1 (TIF 3583 KB)

## Abbreviations

HPVECs: Human placental microvascular endothelial cells
IGF1: Insulin-like growth factor 1
TA: Nanosight tracking analysis
PE: Preeclampsia
TEM: Transmission electron microscopy
