# Supplementary figures and images for: Exosomal miR-486-5p derived from human placental microvascular endothelial cells regulates proliferation and invasion of trophoblasts via targeting IGF1
Source: Hum Cell. 2021 May 11;34(5):1310–23. doi: 10.1007/s13577-021-00543-x (PMC8338855; doi:10.1007/s13577-021-00543-x)

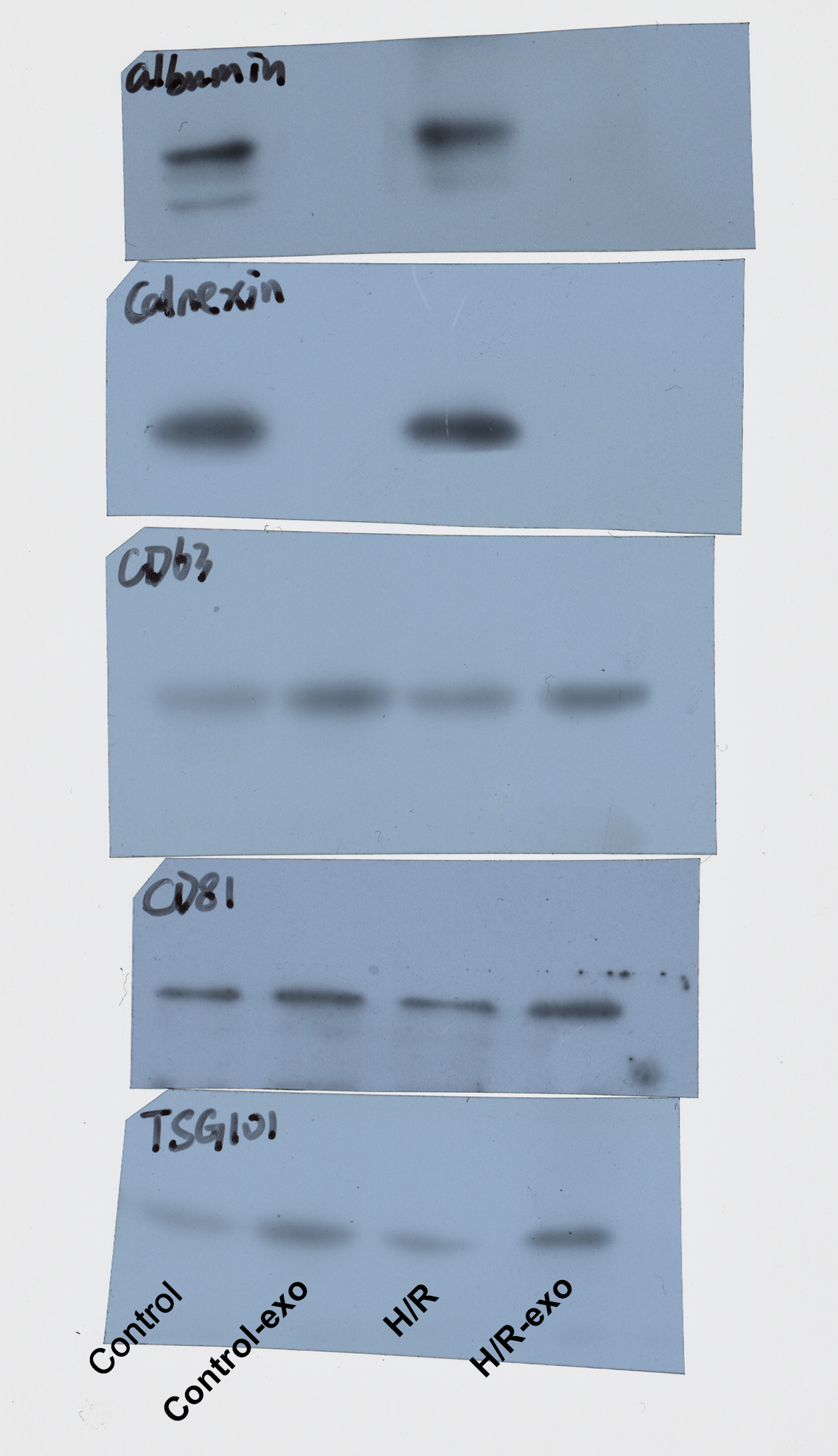

Supplement: Supplementary file 1 — Supplementary file1 (TIF 3583 KB) [file 13577_2021_543_MOESM1_ESM.tif]
